# Work Fatigue in a Non-Deployed Military Setting: Assessment, Prevalence, Predictors, and Outcomes

**DOI:** 10.3390/ijerph16162892

**Published:** 2019-08-13

**Authors:** Michael R. Frone, Ann-Renee Blais

**Affiliations:** 1 Department of Psychology, University at Buffalo, The State University of New York, Buffalo, NY 14203, USA; 2 Department of National Defence, Ottawa, ON K1A 0K2, Canada

**Keywords:** work fatigue, exhaustion, work demands, work resources, personal resources, military morale, workplace cognitive failure, turnover intentions, work–family conflict, military

## Abstract

Although work fatigue represents an important issue among military personnel in combat settings, little attention has been paid to work fatigue in the non-deployed setting. This issue was addressed by (a) validating the Three-Dimensional Work Fatigue Inventory (3D-WFI) among non-deployed military personnel, (b) assessing the prevalence of work fatigue in a non-deployed setting, and (c) exploring several potential predictors and outcomes of work fatigue in this setting. Data came from a large national probability sample (*N* = 1375) of non-deployed Royal Canadian Air Force military personnel. Results demonstrated that the 3D-WFI provided a psychometrically sound assessment of physical, mental, and emotional work fatigue among military personnel, which was invariant across sex, age, military component, and military role. All three types of work fatigue were highly prevalent among military personnel in a non-deployed setting. In terms of predictors, job demands were positively associated, and distributive justice, perceived organizational support, physical activity and sleep quality were negatively associated with each type of work fatigue, whereas role ambiguity was positively associated with mental and emotional work fatigue, and interpersonal justice was negatively associated with physical and emotional fatigue. Abusive supervision and sleep quantity were unrelated to work fatigue. In terms of outcomes, the three types of fatigue were positively associated with workplace cognitive failures and work-to-family conflict. In contrast, mental and emotional work fatigue were negatively related to military morale and positively associated with turnover intentions. This study demonstrates that work fatigue is a critical issue among military personnel in non-deployed settings, and an essential issue for military policy development.

## 1. Introduction

Work fatigue is a critical employee safety and well-being issue for modern military organizations. Much like civilian work environments, militaries have experienced many changes over the past decades that can influence the types and levels of experienced work fatigue [1]. These changes include increases in the number of deployments and 24/7 continuous and sustained operations; increased diversity in mission types (combat, peacekeeping, assisting in natural disasters, delivering humanitarian aid, and nation-building); budget cuts that undermine workforce and material resources required for operational demands; and more cognitively complex work. Although teamwork has always been important in military organizations, there have been increasing interpersonal demands due to greater demographic diversity within the military, as well as expanding requirements to interact with diverse civilian populations [1,2,3,4,5,6,7,8]. The military work environment is varied and complex. Campbell and Nobel [4] identified four broad military work settings by crossing deployment status (non-deployed vs. deployed) and mission type (combat vs. non-combat). (According to Campbell and Noel [4], there are four general military settings that can be identified by crossing deployment status and mission type. Setting 1: The *noncombat, non-deployed setting* represents working and training to maintain personal and unit readiness at one’s home-base in one’s home country. This setting is the focus of the present study. Setting 2: The *noncombat, deployed setting* represents deployment outside one’s home country for such goals as disaster-relief and humanitarian missions. Setting 3: The *deployed, combat setting* is what uniquely defines the military occupation. Military personnel are deployed outside their home country to engage in combat-related activities, such as engaging enemy combatants, policing, or peacekeeping. Setting 4: The *combat, non-deployed setting* represents activities originating from one’s home-base in one’s home country, such as extensive policing or peacekeeping activities.). Unsurprisingly, much military research has focused on the causes and effects of work fatigue, as well as the effectiveness of fatigue countermeasures, within deployed combat settings [1,7,8,9,10,11,12]. Despite the critical importance of such research, it is also vital for military organizations to maintain high levels of performance, workplace safety, positive job attitudes, and physical and mental health among military personnel in the non-combat non-deployed context. 

Our overall goals in this article are to (a) present new and extend prior research on work fatigue in the military and (b) add to the evidentiary base on the Three-Dimensional Work Fatigue Inventory (3D-WFI) [13]. We address these broad goals in several ways. First, we provide the first assessment of the psychometric properties of the 3D-WFI among military personnel working in a non-deployed setting, using national data from the Royal Canadian Air Force (RCAF). Prior military studies have used single-item measures of fatigue developed for specific studies, as well as multi-item measures that do not directly assess fatigue during the workday and have various conceptual limitations. We endeavor to show that the 3D-WFI can provide a valid assessment of work fatigue in the military, thereby providing consistency in the assessment of this construct across military studies. We do this by replicating the factor structure and reliability of the 3D-WFI reported in prior studies of civilian employees in the U.S. [13] and Germany [14]. We also extend these prior psychometric evaluations of the 3D-WFI by providing the first examination of measurement invariance across personal and occupational demographic variables. 

Second, we explore overall and subgroup prevalence of experiencing three types of work fatigue (physical, mental, and emotional) among RCAF military personnel in a non-deployed setting. Estimating the prevalence of deleterious personal outcomes in a population provides essential information on the extent of the problem for the development of future research and evidence-based policy aimed at mitigating its causes and consequences [15,16]. However, to provide reliable and generalizable prevalence estimates, studies must use large samples obtained by formal probability sampling from a well-defined population [15,16]. Documenting the prevalence of work fatigue among military personnel in a non-deployed setting provides essential information for military organizations and the field of occupational safety and health. 

Finally, we use a broader set of predictors and outcomes than seen in prior military research on work fatigue. Campbell and Nobel [4] pointed out that the experience and salience of various work conditions can differ across military work settings, and that the non-combat non-deployed setting is similar to conventional, civilian work settings. Therefore, the development of hypotheses regarding the predictors and outcomes of work fatigue in our military sample draws on theoretical frameworks and empirical research stemming from the broader occupational literature based on civilian workers.

## 2. Assessment of Work Fatigue in the Military

Based on an examination of definitions from the last 100 years, Frone and Tidwell [13] defined work fatigue generally as a subjective experience representing “extreme tiredness and reduced functional capacity that is experienced during and at the end of the workday” (p. 274). (Research often conflates fatigue and sleepiness, such that studies focused on fatigue use measures of sleepiness. However, fatigue and sleepiness represent related, yet distinct constructs [17,18]. Sleepiness represents the desire and tendency to fall asleep, also called sleep propensity, whereas fatigue represents excessive tiredness and reduced functional capacity. In the present article, we do not review studies that did not specifically assess fatigue (i.e., used a measure of sleepiness to assess fatigue). Frone and Tidwell also provided definitions for three types of work fatigue based on the specific energetic resources that are affected [13] (p. 274):

Physical work fatigue represents extreme physical tiredness and reduced capacity to engage in physical activity experienced during and at the end of the workday.

Mental work fatigue represents extreme mental tiredness and reduced capacity to engage in cognitive activity experienced during and at the end of the workday.

Emotional work fatigue represents extreme emotional tiredness and reduced capacity to engage in emotional activity experienced during and at the end of the workday.

As noted earlier, military research has generally focused on fatigue experienced during combat operations [10,11,19]. The experimental simulations often do not assess fatigue, inferring its presence from performance deficits. Also, observational studies of the causes and outcomes of fatigue among deployed personnel use small convenience samples and single-item measures of fatigue developed for specific studies or multi-item measures that do not directly assess fatigue while working. These studies cannot provide direct information on the experience of work fatigue in large populations of military personnel.

Therefore, the present study explores the utility of using the 3D-WFI [13] to assess physical, mental, and emotional work fatigue in a large non-deployed military population. We seek to replicate the basic psychometric properties of this measure—factor structure and reliability— reported in two samples of civilian workers [13,14] using a national probability sample of RCAF military personnel, as well as extend these previous psychometric evaluations by testing measurement invariance across several demographic variables (sex, age, military component, and military role).

## 3. Prevalence of Work Fatigue in a Military Non-Deployed Setting

Knowledge regarding the prevalence and frequency of experiencing work fatigue among military personnel in a non-deployed setting is critical for evaluating the extent to which this population is affected and its salience for military personnel policy. Given the lack of available prevalence data, we take a comprehensive look at the prevalence of all three types of work fatigue in a large probability sample of nondeployed military personnel. Specifically, we explore the following research questions:

Research Question 1 (RQ1): What is the prevalence of experiencing physical, mental, and emotional work fatigue at least monthly, at least weekly, and daily among RCAF personnel in a non-deployed setting?

Research Question 2 (RQ2): Does the prevalence of physical, mental, and emotional work fatigue differ across personal demographics (sex, age), military component (Regular Forces vs. Primary Reserve), and military role (aircrew, maintenance technicians, and other support roles)?

## 4. Predictors and Outcomes of Work Fatigue in a Military Non-Deployed Setting

Because of their interest in fatigue occurring in deployed combat settings, military researchers have focused on a narrow set of potential causes (sleep quantity and quality, circadian disruption due to shiftwork or changes in time zones, and time on task) and outcomes (cognitive and performance errors) of work fatigue [5,7,9,10,11,20]. Although these causes and outcomes are fundamentally important during deployment, they are also crucial in non-deployed settings. Moreover, prior research within the civilian workforce points to other potential causes and outcomes of work fatigue that may be salient in a non-deployed military setting to both military personnel and military leaders. Next, we summarize the theoretical lens used to frame our study of predictors and outcomes of work fatigue.

### 4.1. Theoretical Lens

We develop our hypotheses based on conservation of resources (COR) theory [21,22,23]. According to COR theory, people are motivated to obtain, retain, and protect valued resources. The valued resources at the heart of the present study are personal energies—physical, mental, and emotional. Individuals experience strain (e.g., work fatigue) and consequent strain-related outcomes when they are threatened with resource loss, lose resources, or fail to gain resources following resource investment.

For example, to meet various work demands, individuals need to expend (lose) physical, cognitive, or emotional energy. Thus, job demands lead to an energy depletion process resulting in work fatigue. In contrast, to the extent that individuals experience job and personal resource gain, their energies are protected or renewed, thereby resulting in reductions in work fatigue. Because work fatigue (a form of strain) results from the depletion of and failure to restore energetic resources, it is experienced as an aversive state that undermines a person’s physical, mental, and emotional capacities, subsequently leading to a host of adverse organizational and personal consequences. 

The energy depletion or gain processes described by COR theory that are central to the experience of work fatigue can involve a multitude of predictors (job demands, job resources, personal resources), as well as a multitude of job and personal outcomes. Our goal is to move beyond bivariate associations reported in the previous meta-analyses and studies focused on single predictors or consequences of work fatigue, as well as consider the dimensionality of work fatigue. In doing so, we seek to uncover modifiable predictors that have independent associations with physical, mental, and emotional work fatigue and therefore represent key targets for intervention. The nine predictor variables represent job demands (role overload, role ambiguity, and abusive supervision), job resources (distributive justice, interpersonal justice, and perceived organizational support), and personal resources (physical activity, sleep quantity, and sleep quality). Similarly, to provide a better understanding of associations between work fatigue and outcomes, we explore the simultaneous and independent associations of the three types of work fatigue to four consequences of importance to both military leaders and personnel (military morale, workplace cognitive failures, turnover intentions, and work-to-family conflict). Specific hypotheses involving the predictors and outcomes are developed next.

### 4.2. Predictors of Work Fatigue 

#### 4.2.1. Job Demands

Based on COR theory, chronic exposure to work overload is perhaps the most important source of energetic resource deletion resulting in fatigue during the workday. All work demands require the investment of one or more sources of energetic resources—physical, mental, or emotional. Consistent with this, meta-analytic reviews reported evidence for a bivariate association between overall work overload and overall work exhaustion [24,25]. (These meta-analyses, and other studies cited later, summarize research using the emotional exhaustion (EE) measure from the Maslach Burnout inventory (MBI) [26]. As discussed by Frone and Tidwell [13], the MBI-EE measure (a) does not assess emotional exhaustion distinct from physical or mental exhaustion; (b) it represents an assessment of overall work exhaustion and only one aspect of work fatigue (exhaustion); and (c) it contains items not assessing exhaustion at all (i.e., construct contamination). Because prior research using the MBI-EE measure cannot support or fail to support associations involving emotional fatigue as conceptualized and assessed in this study, we refer to *overall work exhaustion* when summarizing primary studies using or meta-analyses based on the MBI-EE measure.). Because overall work overload represents having too much work to do and an inability to meet expectations, independent of the type of demands (physical, mental, and emotional), we expect that this construct will be positively related to all three types of work fatigue. Therefore, we hypothesize:

**Hypothesis 1** **(H1):** Controlling for all other predictors, work overload will be significantly and positively related to (a) physical, (b) mental, and (c) emotional work fatigue.

Role ambiguity represents another job demand that may cause fatigue at work. Role ambiguity occurs when it is not clear how to reach work goals. Therefore, workers need to expend energetic resources to determine what needs to be done, in addition to the actual completion of tasks, so that there is some performance payoff from the invested energy. Consistent with this argument, a meta-analytic review reported a positive association between role ambiguity and overall work exhaustion [24]. More recently, Frone and Tidwell [13] argued that role ambiguity should be differentially related to the three types of work fatigue. Specifically, because role ambiguity should not deplete physical resources, it will have no significant association with physical work fatigue. In contrast, role ambiguity may deplete mental (i.e., cognitive) resources resulting from the mental effort aimed at trying to determine one’s role expectations and how to meet those expectations. Also, role ambiguity may deplete emotional resources leading to emotional work fatigue due to interpersonal conflicts and stress resulting from uncertainty regarding expectations and responsibilities. However, Frone and Tidwell [13] reported that, after controlling for other predictors, role ambiguity was positively related only to mental work fatigue among U.S. civilian workers. Nonetheless, because of the new population of military personnel, we reexamine Frone and Tidwell’s [13] original hypothesis:

**Hypothesis 2** **(H2):** Controlling for all other predictors, role ambiguity will be significantly and positively related to (a) mental and (b) emotional work fatigue but not significantly related to (c) physical work fatigue.

Because of the inherent hierarchical power structure of organizations, the impact of leaders’ negative behavior on their direct reports in civilian and military organizations is gaining increased attention [27,28,29,30]. Abusive supervision refers to “the extent to which supervisors engage in the sustained display of hostile verbal and nonverbal behaviors, excluding physical contact” [31] ( p. 178). Based on COR theory, exposure to public and private ridicule and undermining by one’s supervisor is a work demand that can deplete emotional energy and increase emotional work fatigue. Although a meta-analysis supports a positive bivariate association between abusive supervision and overall work exhaustion [32], consideration of the specific types of work fatigue can further clarify the association between supervisor abuse and work fatigue. Therefore, we hypothesize:

**Hypothesis 3** **(H3):** Controlling for all other predictors, abusive supervision will be significantly and positively related to (a) emotional work fatigue but not significantly related to (b) physical and (c) mental work fatigue.

#### 4.2.2. Job Resources

COR theory suggests that a negative association exists between job resources and work fatigue because job resources may protect or renew various energies. One potential job resource is organizational justice (i.e., fair treatment at work) In this study, we explore two dimensions of organizational justice—distributive justice and interpersonal justice. Distributive justice represents receiving fair outcomes (e.g., rewards and recognition) given one’s level of input, whereas interpersonal justice represents being treated with respect, dignity, and politeness [33]. Being justly rewarded for one’s efforts and being treated well interpersonally represent a gain in resources. Both sources of justice signal to the individual that they are a valued member of the organization, thereby maintaining or renewing emotional energy. Therefore, the primary impact of experiencing high levels of distributive and interpersonal justice would be to reduce emotional fatigue. It seems less likely that either type of justice would affect physical or mental energies and therefore physical and mental fatigue. Although a study supported a negative association of both distributive and interpersonal justice to overall work exhaustion [34], it cannot speak to the specificity of associations between these two types of justice and the three types of work fatigue. Therefore, we hypothesize:

**Hypothesis 4** **(H4):** Controlling for all other predictors, distributive justice will be significantly and negatively related to (a) emotional work fatigue but not significantly related to (b) physical and (c) mental work fatigue.

**Hypothesis 5** **(H5):** Controlling for all other predictors, interpersonal justice will be significantly and negatively related to (a) emotional work fatigue but not significantly related to (b) physical and (c) mental work fatigue.

The perceived supportiveness of a work organization may represent an important work-related resource that can affect levels of work fatigue. Perceived organizational support represents the extent to which employees believe that “the organization values their contributions and cares about their well-being” [35] (p. 1855). This perception partly results from experiencing favorable treatment by the organization, receiving support and help from supervisors and coworkers, and receiving the organizational resources required to perform one’s job successfully. COR theory would suggest that the socioemotional and task-related resources resulting in perceptions of organizational support should lead to reductions in all three types of work fatigue. Although no research has tested this proposition, Kurtessis et al.’s [35] meta-analysis found a significant negative correlation between perceived organizational support and overall work exhaustion. Based on the reasoning above, and considering the dimensionality of work fatigue, we hypothesize:

**Hypothesis 6** **(H6):** Controlling for all other predictors, perceived organizational support will be significantly and negatively related to (a) physical, (b) mental, and (c) emotional work fatigue.

#### 4.2.3. Personal Resources

In addition to job resources, COR theory suggests that a negative association may exist between personal resources and work fatigue because personal resources may renew or protect various energies. One relevant personal resource is physical activity. Research shows that physical activity improves physical health and physiological fitness [36,37,38], cognitive functioning [38,39,40], and emotional functioning [36,37,38]. As a result, physical activity may increase the reservoir of physical, mental, and emotional energies. This increased energy allows individuals who are physically active to meet physical, mental, and emotional work-related demands with lower levels of associated fatigue than individuals who are not physically active. Consistent with this argument, several studies support a negative association between physical activity and overall work exhaustion [41,42,43]. However, it is not clear from these studies whether the potential impact of physical activity on work fatigue is general, affecting all three types of fatigue, or is specific to physical fatigue. Assuming the effect of physical activity on work fatigue is more general, we hypothesize:

**Hypothesis 7** **(H7):** Controlling for all other predictors, level of physical activity will be significantly and negatively related to (a) physical, (b) mental, and (c) emotional work fatigue.

Based on COR theory, sleep represents an essential personal resource that may reduce and protect one from experiencing fatigue at work. Although the purpose served by sleep is poorly understood and many explanatory models exist [44], energy conservation and restoration represent a possible function of sleep [44,45,46,47]. Sleep can be characterized along two dimensions—quantity (duration) and quality [48]. Sleep quantity represents the number of hours of uninterrupted sleep and sleep quality refers to how well one sleeps. Both longer duration and higher quality sleep should result in better energy restoration and reduced worker fatigue. Supporting this view, research has documented a negative association between sleep quality and overall work exhaustion [49,50,51]. However, one study looking at sleep quantity and overall work exhaustion failed to find an association [52]. We extend this body of research by exploring the simultaneous associations of sleep quantity and sleep quality to the three types of work fatigue. Based on this discussion, we hypothesize:

**Hypothesis 8** **(H8):** Controlling for all other predictors, sleep quantity will be significantly and negatively related to (a) physical, (b) mental, and (c) emotional work fatigue.

**Hypothesis 9** **(H9):** Controlling for all other predictors, sleep quality will be significantly and negatively related to (a) physical, (b) mental, and (c) emotional work fatigue.

### 4.3. Outcomes of Work Fatigue

#### 4.3.1. Military Morale

Military morale represents a motivational construct that can impact performance [53] and is defined as “motivation and enthusiasm to perform well within a specified context” [54] (p. 35). Although military morale has been primarily a concern within in the context of military operations during deployment, it is also essential in non-deployed settings [53,54]. We expect that the extreme tiredness and reduced functional capacity that compose work fatigue can undermine an individual’s motivation and enthusiasm to perform well. We are not aware of any research linking work fatigue to military morale. Based on the expectation that all three types of work fatigue might undermine military morale, we hypothesize:

**Hypothesis 10** **(H10):** When considered simultaneously, (a) physical, (b) mental, and (c) emotional work fatigue will be significantly and negatively related to military morale.

#### 4.3.2. Workplace Cognitive Failure

Cognitive failures at work represent a potentially critical proximal cause of performance errors and safety incidents [55]. Wallace and Chen [55] defined workplace cognitive failures as lapses while working in memory (failure to retrieve relevant work-related information), attention (failure to focus attention on task-relevant information), and action (failure to execute appropriate and intended behaviors and actions while working). Research suggests that workplace cognitive errors are associated with job performance [56], as well as lower safety compliance, more unsafe behaviors, and a higher likelihood of being injured at work [55,57].

Although we are not aware of research examining the association between work fatigue and workplace cognitive failures, there is reason to expect an association. Wallace and Chen [55] suggested that workplace cognitive failures result from a self-regulatory failure allowing off-task attention to interfere with or replace on-task attention. Also, and consistent with COR theory, Boksem and Tops [58] (p. 131) proposed that “fatigue corresponds to a drive to abandon behavior when energetical costs exceed perceived benefits of continued performance.” Therefore, the extreme tiredness and reduced functional capacity that compose work fatigue may result in an aversion to current on-task activity, thereby allowing attention to drift to off-task activities that present potentially higher perceived benefits to the continued expenditure of energy. Although it might seem reasonable to suspect that only mental work fatigue would be related to workplace cognitive failures, the execution of all tasks requiring physical, mental, or emotional energy is cognitively mediated, requiring memory, attention, and action. Therefore, we expect that all three sources of work fatigue can affect workplace cognitive failures, and consequently hypothesize:

**Hypothesis 11** **(H11):** When considered simultaneously, (a) physical, (b) mental, and (c) emotional work fatigue will be significantly and positively related to workplace cognitive failures.

#### 4.3.3. Turnover Intentions

Based on COR theory, the frequent experience of work fatigue is physically and psychologically aversive, which would motivate an individual to escape the situation. Consistent with this expectation, a meta-analytic review reported that overall work exhaustion was positively related to turnover intentions [24]. Using the 3D-WFI, Frone and Tidwell [13] reported the only study to explore the associations of the three types of fatigue to turnover intentions. These researchers found that among U.S. civilian workers, physical and emotional work fatigue were positively associated with turnover intentions, though the association was not significant for mental work fatigue. Despite these results, it seems plausible that frequent exposure to chronic mental work fatigue can motivate a desire to leave a job. Therefore, we reexamine Frone and Tidwell’s [13] original hypothesis among military personnel:

**Hypothesis 12** **(H12):** When considered simultaneously, (a) physical, (b) mental, and (c) emotional work fatigue will be significantly and positively related to turnover intentions.

#### 4.3.4. Work-to-Family Conflict

Work and family represent two important social roles that compose the lives of most individuals [59]. One result of holding both work and family roles is the experience of work–family conflict, where work and family roles can interfere with each other [60]. In the present study, we focus on work-to-family conflict, where meeting the demands and responsibilities of one’s work role interferes with meeting the obligations of one’s family role. Although most research on work-to-family conflict has focused on civilian workers, Britt and Dawson [61] noted that the negative impact of military demands on family life have not gone unnoticed in the military, and that “even when soldiers are in garrison, the level of workload places demands on even the healthiest of families” (p. 204).

The frequent depletion of energetic resources during the workday should undermine an individual’s motivation and ability to meet obligations at home, leading to reports of more frequent work-to-family conflict. There is some general support for this assertion in civilian worker samples, where longitudinal research shows that baseline assessments of overall work exhaustion predict higher levels of subsequent work-to-family conflict [62,63]. However, no research has explored whether work-to-family conflict is associated with depletion of a specific energetic resource, or the depletion of all three energetic resources at work. Moreover, no research has examined the association between work fatigue and work-to-family conflict in military populations. Because physical, mental, and emotional energy are each essential to meeting the varied demands and responsibilities composing the family role, we hypothesize:

**Hypothesis 13** **(H13):** When considered simultaneously, (a) physical, (b) mental, and (c) emotional work fatigue will be significantly and positively related to work-to-family conflict.

## 5. Materials and Methods 

### 5.1. Study Design

A stratified random sample of the target population (i.e., RCAF personnel in a non-deployed setting) was selected from a sampling frame (i.e., a total of 16,010 military and civilian personnel) available via the Defence Resource Management Information System. This sampling frame was stratified into six organizations covering the 1st Canadian Air Division and the 2nd Canadian Air Division. Random samples were drawn from each of the six strata with proportional allocation for military component (i.e., Regular Force, Primary Reserve, and civilian personnel), sex, rank (i.e., non-commissioned members (NCMs) and officers) for military personnel, and years of service for civilian personnel. This proportional allocation increased the probability of proper representation of survey respondents on these variables. The random samples yielded a total sample of 5263 RCAF personnel (after necessary exclusions, such as undeliverable emails).

The survey administration received approval from the Canadian Armed Forces (CAF) Social Science Research Review Board. The 5263 sampled personnel were invited to participate in the Defence Workplace Wellbeing Survey/Director Flight Safety—Fatigue Questionnaire (DWWS/DFS-FQ) via email or postcards, and the online survey was live from May to August 2018. This process resulted in 2059 respondents completing the DWWS/DFS-FQ, for an overall response rate of approximately 39%. The respondents provided informed consent and were assured that only aggregate data would be reported. We restricted the analyses to the 1375 Regular Force and Primary Reserve members (i.e., excluding civilian employees) of the RCAF who completed the English version of the DWWS/DFS-FQ and had no missing data on the demographic variables used in this study. 

### 5.2. Sampling Weights

Respondents within each of the six organizations were post-stratified by military component and rank group (i.e., junior NCM, senior NCM, junior officer, and senior officer). Sampling weights were calculated so that respondents would represent the target population on the original stratification variable (i.e., organization) and post-stratification variables (i.e., military component and rank group). All reported descriptive and inferential statistics are weighted using the sampling weights.

### 5.3. Respondent Characteristics

We describe the population characteristics with weighted percentages. Eighty-six percent of RCAF members were male. Thirty-five percent were younger than 35 years old, 55% were 35 to 54 years old, and 10% were 55 years or older. Eighty-seven percent of RCAF members belonged to the Regular Force and 13% to the Primary Reserve. Also, 57% were junior NCMs, 20% were senior NCMs, 16% were junior officers, and 7% were senior officers. Thirty-three percent of RCAF members had served 1 to 10 years in the RCAF, 35% had served 11 to 19 years, and 32% had served 20 or more years. In terms of military role in the RCAF, 18% of were aircrew, 37% were maintenance technicians, and 45% occupied other support roles (e.g., clerk, cook, and firefighter). In the past month, 74% of RCAF members had predominantly worked straight days, less than 1% had predominantly worked straight nights, and 26% had predominantly worked variable shifts.

### 5.4. Measures

#### 5.4.1. Work Fatigue

The 18-item Three-Dimensional Work Fatigue Inventory (3D-WFI) [13] was used to assess physical, mental, and emotional work fatigue. The full measure can be viewed in the appendix to an open access accepted manuscript of the Frone and Tidwell article [13] at https://www.ncbi.nlm.nih.gov/pmc/articles/PMC4505929. Each type of work fatigue was assessed with a commensurately worded set of six items with three items assessing extreme tiredness and three items assessing reduced functional capacity. The reporting period in this study was the past month rather than the past 12 months used in the original measure. All items used a 5-point frequency response scale ranging from (1) Everyday to (5) Never. Before averaging across items to compute an overall score for each dimension of work fatigue, we reverse scored the items so that higher scores represented higher levels of fatigue. In the current study, the internal consistency reliability estimates were 0.97 for physical work fatigue, 0.98 for mental work fatigue, and 0.99 for emotional work fatigue.

In order to assess the reported prevalence of each type of work fatigue by the frequency of experiencing it, we created three dichotomous variables for each type of fatigue. We scored the prevalence of at least monthly work fatigue such that 0 = mean score ≤2.5 and 1 = mean score >2.5. We scored the prevalence of at least weekly work fatigue such that 0 = mean score ≤3.5 and 1 = mean score >3.5. Lastly, we scored the prevalence of daily work fatigue such that 0 = mean score ≤4.5 and 1 = mean score >4.5.

#### 5.4.2. Role Overload

The six-item Reilly Role Overload Scale [64] was used to measure this construct. All items used a 7-point frequency response scale ranging from (1) Never to (7) Always. A sample item is “I cannot ever seem to catch up.” The internal consistency reliability estimate was 0.93.

#### 5.4.3. Role Ambiguity

Role Ambiguity was assessed with the six-item role ambiguity subscale of the Role Stressor Scale [65]. All items used a 7-point response scale ranging from (1) Strongly Disagree to (7) Strongly Agree. A sample item is “I am not sure what is expected of me at work.” The internal consistency reliability estimate was 0.91. 

#### 5.4.4. Abusive Supervision

Mitchell and Ambrose’s [66] shortened five-item version of Tepper’s [31] Abusive Supervision measure was used to assess this construct. All items used a 7-point response scale ranging from (1) Strongly Disagree to (7) Strongly Agree. A sample item is “My supervisor ridicules me.” The internal consistency reliability estimate was 0.94.

#### 5.4.5. Distributive Justice

The four-item Contingent Reward subscale of the Job Satisfaction Survey [67] was used to measure distributive justice. All items used a 6-point response scale ranging from (1) Disagree Very Much to (6) Agree Very Much. A sample item is “I don’t feel my efforts are rewarded the way they should be.” The internal consistency reliability estimate was 0.84.

#### 5.4.6. Interpersonal Justice

This construct was assessed with the four-item interpersonal justice subscale of the Organizational Justice Scale [33]. All items used a 5-point response scale ranging from (1) To a Very Small Extent to (5) To a Very Large Extent. A sample item is “Please indicate the extent to which individuals (coworkers, supervisors, etc.) treat you with respect.” The internal consistency reliability estimate was 0.93. 

#### 5.4.7. Perceived Organizational Support

Eight items from the Perceived Organizational Support scale [68] were used to measure this construct. All items used a 7-point response scale ranging from (1) Strongly Disagree to (7) Strongly Agree. A sample item is “Help is available from the organization when I have a problem.” The internal consistency reliability estimate was 0.92.

#### 5.4.8. Physical Activity

The four-item Concise Physical Activity Questionnaire [69] was used to measure this construct. All items used a 5-point frequency response scale ranging from (0) Physically unable or not medically allowed to do this/Chose not to do this to (4) 6–7 days per week. A sample item is “In the past month, indicate how many days per week you engaged in moderate aerobic activity (e.g., brisk walking, bicycling, tennis) for at least 20 consecutive minutes.” The internal consistency reliability estimate was 0.73.

#### 5.4.9. Sleep Quantity

The quantity of sleep was assessed with the following single item: During the past month, how much uninterrupted sleep did you manage to achieve each night (or day for shift workers) on average? This item used a response scale ranging from 0 to 24 hours. 

#### 5.4.10. Sleep Quality

Sleep quality was measured with the following single item: “During the past month, how many nights (or days for shift workers) did you experience poor quality sleep?” This item used a response scale ranging from 0 to 31. We reverse scored this measure so that higher values represented better sleep quality.

#### 5.4.11. Military Morale

This construct was assessed with the six-item Military Morale Scale [53], adapted for use in non-deployed settings [70]. All items used a 5-point frequency response scale ranging from (1) Very Low to (5) Very High. A sample item is “Your level of morale.” The internal consistency reliability estimate was 0.95 in this sample.

#### 5.4.12. Workplace Cognitive Failure

The 15-item measure developed by Wallace and Chen [55] was used to measure this construct. All items used a 5-point frequency response scale ranging from (1) Never to (5) Very Often. A sample item is “Did not focus your full attention on work activities.” The internal consistency reliability estimate was 0.90.

#### 5.4.13. Turnover Intentions

This construct was assessed with the three-item Turnover Intentions Scale [71]. All items used a 5-point response scale ranging from (1) Strongly Disagree to (5) Strongly Agree. A sample item is “I frequently think of quitting my job.” The internal consistency reliability estimate was 0.81. 

#### 5.4.14. Work-to-Family Conflict

This construct was assessed with Netemeyer, Boles, and McMurrian’s five-item work-to-family conflict measure [72]. All items used a 7-point response scale ranging from (1) Strongly Disagree to (7) Strongly Agree. A sample item is “The demands of my work interfere with my home and family life.” The internal consistency reliability estimate was 0.95. 

### 5.5. Statitical Analyses

#### 5.5.1. Confirmatory Factor Analyses and Measurement Invariance

We used single-group and multiple-group confirmatory factor analysis (MG-CFA) with M*plus* (Version 8) [73] and its robust maximum likelihood estimator to verify the fit of the a-priori correlated, three-factor (physical, mental, and emotional work fatigue) model with 18 design-driven correlated errors underlying the 3D-WFI (for more detail, see [13]). Also, to address item-level missing data, we relied on M*plus*’ full information maximum likelihood procedure. We evaluated model fit and tests of measurement invariance for the 3D-WFI across several demographic groups (sex, age group, military component, and military role). We considered five levels of measurement invariance (MI), which collectively assess the equivalence of physical, mental, and emotional fatigue measures across groups composing each demographic variable used in this study (e.g., males and females for respondent sex). First, configural invariance is tested by simultaneously estimating the hypothesized factor structure across the multiple groups with all estimated parameters free to vary across groups. Support for configural invariance indicates that the basic factor structure is the same in each group, and is required before testing other levels of MI. Second, weak invariance is evaluated by constraining the item loadings to be equal across the groups. Support for weak invariance indicates similar item loadings across the groups, which means that the factors have the same meaning in each group. Third, strong invariance is evaluated by constraining the item loadings and intercepts to be equal across the groups. Support for strong invariance indicates similar item intecepts across the groups, which allows comparison of latent means across the groups. Fourth, strict invariance is evaluated by constraining the item loadings, intercepts, and item residual variances to be equal across the groups. Support for strict invariance indicates that the item residual variances are similar across the groups, which allows comparison of observed means (and prevalence rates) across the groups. Fifth, because structural correlations among the residuals of parallel worded items were expected [13,14], we tested a final level of strict + correlated residual invariance by constraining the item loadings, intercepts, residual variances, and select covariances among residuals to be equal across groups. Although this last level of invariance involving correlated residuals is not substantively important, it provides for a complete test of measurement invariance for the 3D-WFI.

To assess overall model fit, we report the Satorra–Bentler (S-B) scaled chi-square with its degrees of freedom (*df*) and correction factor (CF). However, because this statistic can be overly sensitive to large samples [74,75], we also report the following approximate fit indices: (a) the root mean square error of approximation (RMSEA) and its 90% confidence interval, (b) the comparative fit index (CFI), and (c) the standardized root mean square residual (SRMR). Excellent model fit is suggested when RMSEA ≤ 0.06, CFI ≥ 0.95, and SRMR ≤ 0.08, and acceptable model fit is suggested when RMSEA ≤ 0.08, CFI ≥ 0.90, and SRMR ≤ 0.10 [76]. Because several subgroups have relatively small samples, it should be noted that Rigdon [77] demonstrated that when the sample size is small (i.e., less than 300), the RMSEA will reject a model that otherwise demonstrates acceptable fit using the CFI, and this rejection becomes more severe with decreasing sample size. Therefore, for small subgroups, evaluation of single-group model fit should focus on the CFI.

Finally, to evaluate change in model fit between the nested measurement invariance models, given the chi-square difference test’s sensitivity to trivial differences and fluctuations in the context of invariance testing [78], using change in CFI values is recommended [74,78,79]. Increasing levels of invariance are supported if ΔCFI < −0.01.

#### 5.5.2. Predictor and Outcome Regression Analyses

Consistent with prior reports investigating the construct validity of the 3D-WFI among civilian workers, we report multiple linear regression analyses [13,14]. Although we also show zero-order correlations, we focus on the regression results for the tests of hypotheses, because they provide more nuanced information on the independent associations of the predictors to work fatigue and the associations of the three types of work fatigue to the outcome variables. For the predictor relations, we regressed each type of work fatigue on all nine predictor variables simultaneously. For the outcome relations, we regressed each outcome variable on the three types of work fatigue simultaneously. We conducted the linear regressions, using sampling weights, with M*plus* (Version 8) [73]. We addressed missing data at the construct level with M*plus*’ procedure for multiple imputations using Bayesian analysis to create 20 multiply imputed data sets.

## 6. Results

Table 1 provides the weighted descriptive statistics for and correlations among the study variables.

### 6.1. Confirmatory Factor Analyses and Measurement Invariance

#### 6.1.1. Overall Sample

The fit indices for the three-factor model with correlated design-driven residuals was excellent, χ2(114) = 668.88, *p* < 0.001, RMSEA [90% CI] = 0.059 [0.055, 0.064], CFI = 0.96, SRMR = 0.026. All items loaded significantly (all *p*-values < 0.001) and highly on their respective fatigue factor. Standardized factor loadings ranged from 0.87 to 0.96 (mean = 0.91) for physical work fatigue, 0.92 to 0.97 (mean = 0.94) for mental work fatigue, and 0.92 to 0.98 (mean = 0.96) for emotional work fatigue. The internal consistency reliability estimates were 0.97 for physical work fatigue, 0.98 for mental work fatigue, and 0.99 for emotional work fatigue. 

#### 6.1.2. Subgroups and Measurement Invariance

Overall model fit for the various individual subgroups, tests of model fit for all measurement invariance tests, and changes in fit across invariance models are reported in Table 2. The three-factor solution showed at least an acceptable fit to the data in each of the individual subgroups. Moreover, for sex, age, military component, and military role, the results support configural invariance, with all RMSEAs ≤ 0.072, CFIs ≥ 0.953, and SRMRs ≤ 0.031). Finally, for sex, age, military component, and military role, the tests of measurement invariance support weak, strong, strict, and strict + correlated residuals invariance (all ΔCFIs ≤ −0.002). Overall, these results show that the 3D-WFI provides an assessment of physical, mental, and emotional fatigue that is equivalent across the variables mentioned above. 

### 6.2. Prevalence of Work Fatigue

To address RQ1, we report the prevalence of experiencing physical, mental, and emotional work fatigue at least monthly, at least weekly, and daily among nondeployed RCAF military personnel (see the Measures section for the cut-offs used). The prevalence rates reported in Table 3 for the overall sample show three salient patterns. First, a high percentage of RCAF personnel report experiencing all three types of work fatigue at least monthly and at least weekly, and even the percentage of individuals reporting the daily experience of each type of fatigue is notable. Second, the prevalence of experiencing physical and mental work fatigue was similar and higher than the prevalence of experiencing emotional work fatigue. Third, these two general trends across types of fatigue were observed for each of the subgroups. 

To investigate RQ2, we compared each of the prevalence estimates across personal demographics (i.e., sex, age group), military component (i.e., Regular Forces vs. Primary Reserve), and military role (i.e., aircrew, maintenance technicians, and other support roles). In terms of sex differences, there was a tendency for women to report higher levels of all three types of work fatigue than men, though significant differences were found only for daily physical fatigue, ≥weekly mental fatigue, and ≥monthly and ≥weekly emotional fatigue. The results show a general trend where the prevalence of all three types of work fatigue and at each level of frequency of experience was lower among the oldest age group (55 and over) compared with the other two age groups, which did not differ from each other in the prevalence of work fatigue. Members of the Regular Force consistently reported a higher prevalence of all three types of work fatigue and at each level of frequency of experience compared with members of the Primary Reserve. Finally, the prevalence of all three types of work fatigue, regardless of the frequency of experiencing fatigue, was similar across different RCAF military roles.

### 6.3. Predictors of Work Fatigue

We report the associations between the predictor variables and the three types of work fatigue in Table 4. Regarding the work demands, work overload was significantly and positively associated with all three types of work fatigue, thereby supporting H1. Role ambiguity was significantly and positively related to both mental and emotional fatigue but not to physical work fatigue, thereby supporting H2. Finally, H3 received partial support. Counter to prediction, abusive supervision was not significantly associated with emotional work fatigue, although, as expected, it was not associated with physical and mental work fatigue.

Among the work resources, distributive justice was significantly and negatively related to all three types of work fatigue. These results partially support H4 concerning the predicted association between distributive justice and emotional work fatigue, though the significant associations between distributive justice and both physical and mental fatigue were not expected. Interpersonal justice was significantly and negatively associated with physical and emotional work fatigue but not with mental work fatigue. These results provide partial support for H5, because the significant association between interpersonal justice and physical fatigue was not predicted. Perceived organizational support was significantly and negatively related to all three types of work fatigue, thereby supporting H6.

Among the personal resources, physical activity was significantly and negatively associated with all three types of work fatigue, thereby supporting H7. Sleep quantity was not significantly associated with all three types of fatigue, failing to support H8. Finally, sleep quality was significantly and negatively associated with all three types of work fatigue, thereby supporting H9.

### 6.4. Outcomes of Work Fatigue

We report the associations between the three types of work fatigue and the outcome variables in Table 5. Mental and emotional work fatigue, but not physical work fatigue, were significantly and negatively related to morale, thereby providing partial support for H10. All three types of work fatigue were significantly and positively related to workplace cognitive failure, thereby supporting H11. Mental and emotional fatigue, but not physical work fatigue, were significantly and positively associated with turnover intentions, thereby partially supporting H12. Finally, all three types of work fatigue were significantly and positively related to work-to-family conflict, thereby supporting H13.

## 7. Discussion

Work fatigue is a vital issue for workplace safety, job attitudes, health, and performance. Our general goal was to provide the first detailed examination of work fatigue in a large population of non-deployed military personnel. Specifically, given the lack of validated measures to assess multiple dimensions of work fatigue in this setting, we examined whether the 3D-WFI would provide a psychometrically sound and valid measure among military personnel. We also provide the first comprehensive assessment of the prevalence of work fatigue in a large military population in a non-deployed setting. Finally, as part of our validation efforts, building from COR theory and prior civilian research, we explored a broad set of modifiable predictors and potential outcomes of work fatigue among nondeployed military personnel.

### 7.1. Assessment of Work Fatigue in the Military

The present results replicate and extend two prior psychometric evaluations of the 3D-WFI in U.S. and German civilian work populations [13,14]. Consistent with prior research among civilian workers, confirmatory factor analysis supported the three-dimensional structure assessing physical, mental, and emotional work fatigue and internal consistency reliability estimates were uniformly high across the three types of work fatigue. Moreover, this is the first study to demonstrate that the factor structure of the 3D-WFI shows at least an acceptable fit in various subgroups defined by sex and age, as well as military component (Regular Force and Primary Reserve) and military role (aircrew, maintenance technicians, and other support roles) in the RCAF. The results also revealed that the 3D-WFI evinced configural, weak, strong, strict, and strict + correlated residual invariance across these four demographic variables. Our findings suggest that the 3D-WFI provides a psychometrically sound assessment of work fatigue in a military population, and can increase the comparability of results across future studies of military personnel. Nonetheless, the development of a brief version of the 3D-WFI would be useful for research on military personnel during deployment, especially in combat settings.

### 7.2. Prevalence of Work Fatigue in the Non-Deployed Military Setting

This study presents the first broad attempt to examine the prevalence of work fatigue in a large military population. Our findings suggest that physical, mental, and emotional work fatigue are highly prevalent among non-deployed RCAF military personnel. Approximately 62% report experiencing both physical and mental fatigue ≥monthly, 42% report experiencing both types of work fatigue ≥weekly, and 14% report experience both types of work fatigue daily. Even though emotional work fatigue was less prevalent than physical and mental work fatigue, it was still highly prevalent, with approximately 43% experiencing emotional work fatigue ≥monthly, 29% experiencing it ≥weekly, and 9% report experiencing it daily. 

No published prevalence data exist using the 3D-WFI in a national sample of civilian workers to view as a comparison. However, we were able to assess the prevalence of work fatigue with the 3D-WFI using Frone and Tidwell’s [13] national data on U.S. wage and salary workers. The prevalence rates for physical (56% ≥monthly, 29% ≥weekly, 7% daily), mental (56% ≥monthly, 28% ≥weekly, 6% daily), and emotional (35% ≥monthly, 15% ≥weekly, 3% daily) work fatigue among U.S. civilian workers show two relevant patterns. First, consistent with non-deployed RCAF military personnel, physical and mental work fatigue showed similar prevalence rates and both were more prevalent than emotional work fatigue. Second, and more importantly, the prevalence of all three types of work fatigue among civilian workers was substantially lower than the prevalence among non-deployed RCAF military personnel, especially as the frequency of experiencing work fatigue increased. 

Collectively, the present results and those from Frone and Tidwell’s [13] data suggest that the prevalence of work fatigue represents a critical issue for both civilian workers and military personnel, though this issue is especially prominent in the military. The prevalence of at least weekly and daily work fatigue in the non-deployed RCAF military population should be of concern to military leaders and provide a strong rationale for policy development and further research on work fatigue in this setting.

Turning to demographic differences in the prevalence of work fatigue, there is little evidence for consistent significant sex differences or any differences across military roles (aircrew, maintenance technicians, and other support roles). There was evidence that older military personnel (55 and older) reported lower levels of all three types of fatigue than younger personnel (18–35 and 35–55). Although this finding might seem surprising, compared with younger personnel, it may be that older personnel have overlearned their primary work responsibilities, thereby requiring less energy to complete them. Also, older personnel may have discovered ways to complete their assignments while minimizing depletion of energetic resources or are more able to secure institutional resources that help minimize depletion of physical, mental, and emotional energies. Finally, the results showed that the prevalence of each type of work fatigue was substantially higher among the Regular Force than among those in the Primary Reserve. This difference may be due to several factors: (a) dissimilarities in the work assignments of Regular Force and Primary Reserve members, (b) differences in employment status (i.e., full-time vs. part-time), and (c) differences in work contracts or psychological attachments to the military that may allow reservists to experience less fatigue or more readily leave the military if experiencing excessive work fatigue. Future research will need to explore the reasons for these observed age and military component differences in the prevalence of work fatigue. 

### 7.3. Predictors of Work Fatigue

This study explored the simultaneous associations of three work demands (role overload, role ambiguity, abusive supervision), three job resources (distributive justice, interpersonal justice, perceived organizational support), and three personal resources (physical activity, sleep quantity, sleep quality) to each type of work fatigue. Our results show that all three groups of variables were associated with work fatigue. Among job demands, role overload was positively associated with each type of work fatigue, whereas role ambiguity was positively associated with the psychologically based types of work fatigue (i.e., mental and emotional). Similar to a previous meta-analysis [32] reporting a positive bivariate association between abusive supervision and overall work exhaustion, this study also supported positive bivariate associations between abusive supervision and each type of work fatigue. However, our regression analyses showed that when adjusting for other predictors, abusive supervision is not independently associated with any dimension of work fatigue. Abusive supervision may be directly associated with many deleterious outcomes [30,32], but depletion of physical, mental, and emotional resources may not be among them. However, future longitudinal research using a broad set of potential causes of physical, mental, and emotional work fatigue will need to confirm the present findings. 

Among the job resources, distributive justice was negatively associated with all three types of work fatigue, whereas interpersonal justice was negatively associated with physical and emotional fatigue. We expected that these two types of organizational justice would be negatively associated with emotional work fatigue, which our data confirmed. However, the negative associations with physical and mental work fatigue were unexpected. This finding suggests that fair treatment in terms of rewards and during interpersonal interactions may have a broader protective association with work fatigue. Perceived organizational support is a potentially critical predictor of energetic resources. Our findings support this expectation by showing that perceived organizational support was negatively associated with all three dimensions of work fatigue. 

Finally, personal resources were also important predictors of work fatigue. Physical activity was negatively associated with all three dimensions of work fatigue. These results suggest that physical activity may increase the reservoir of physical, mental, and emotional energies, which allows individuals to meet physical, mental, and emotional work-related demands with lower levels of associated fatigue than individuals who are not physically active. 

Although past research on sleep and overall work exhaustion did not simultaneously examine both sleep quantity and sleep quality, our results extend this body of research by showing more directly that sleep quality [49,50,51], but not sleep quantity [52], is negatively associated with all three types of work fatigue. Therefore, our results suggest that sleep quality represents a vital physiological process that may conserve and restore physical, mental, and emotional energy, which respectively reduces the experience of physical, mental, and emotional fatigue at work.

Overall, the present findings underscore the need for future research to consider the multidimensional nature of work fatigue, and the fact that work fatigue may be caused by multiple variables inside the workplace and multiple personal behaviors. Nonetheless, future research should explore other variables that fall within the three types of predictors, and also explore various dimensions of personal (i.e., nonwork) demands, such as financial and family problems. 

### 7.4. Outcomes of Work Fatigue

We examined a broad set of potential outcomes and found independent associations involving all three types of work fatigue. In terms of personal outcomes, physical, mental, and emotional fatigue were each independently and positively associated with more frequent work-to-family conflict. Although prior research found that overall work exhaustion was positively related to work-to-family conflict [62,63], the present results extend this prior finding by suggesting that frequent depletion of all three energetic resources may undermine one’s ability to meet family demands and responsibilities.

Turning to the organizational outcomes, military morale, workplace cognitive failures, and turnover intentions were primarily associated with mental and emotional work fatigue. Only workplace cognitive failure was associated with physical work fatigue. The results for turnover intentions partly support one prior study of civilian workers looking at all three types of work fatigue. Consistent with the present study, Frone and Tidwell [13] found a positive association between emotional work fatigue and turnover intentions. However, Frone and Tidwell found that among civilian workers physical fatigue was associated with turnover intentions, whereas the present study of non-deployed military personnel found that mental fatigue was associated with turnover intentions. This difference might be due to the different populations in the two studies (civilian workers vs. military personnel), though the exact mechanism causing the difference is unclear and an issue for future research. Being the first study to explore the association of work fatigue to both military morale and workplace cognitive failures, this study extends prior research on the potential outcomes of work fatigue. Both outcomes are primarily associated with the frequent depletion of psychological (mental and emotional) energetic resources, though physical fatigue also may play a role in workplace cognitive failures.

Overall, the present findings show that the frequent depletion of all three types of energetic resources is associated with a broad set of adverse outcomes. Therefore, future research on the outcomes of work fatigue should consider the multidimensional nature of work fatigue. Future research also could expand the set of outcomes used in this study to include direct assessments of task and contextual job performance [80], as well as safety compliance and participation, safety events, and injuries [57,81]. 

### 7.5. Practical Implications

Our findings show that all three types of work fatigue are highly prevalent among non-deployed RCAF military personnel. The results also show that mental and emotional fatigue were independently associated with poorer scores on all employee outcomes, and physical work fatigue was independently associated with workplace cognitive failures and work-to-family conflict. Taken together, these findings suggest that work fatigue in a non-deployed setting represents an important issue for military organizations and military personnel. In terms of prevention efforts, our findings from several modifiable risk and protective factors suggest that all three types of work fatigue may be multi-determined. Therefore, multicomponent prevention interventions should target multiple potential causes of work fatigue. At the organization level, intervention efforts need to reduce exposure to work overload and role ambiguity, as well as increase organization justice (fair treatment in terms of rewards and interpersonal interactions) and organizational support. At the person level, intervention efforts need to help individuals learn to manage more effectively risk exposures and need to promote positive personal behaviors that may reduce work fatigue, such as increasing the frequency of physical activity at and away from work and increasing sleep quality. Developing a well-crafted, integrated multicomponent intervention may take longer to develop, but may be more effective than focusing on a single modifiable cause. 

### 7.6. Study Strengths and Limitations

The present results should be considered within the context of the strengths and limitations of this study. A strength of this study was that it utilized a large probability sample of non-deployed RCAF military personnel. Large probability samples provide: (a) more variation in the predictors and outcome variables; (b) adequate statistical power to detect hypothesized effects; and (c) more accurate estimates of population parameters [82,83,84]. The present sample also increases the generalizability of the results to the population of interest; in this case, RCAF military personnel in a non-deployed setting. 

These strengths notwithstanding, four study limitations should be considered. First, a cross-sectional design was used, which limits inferences regarding the existence and direction of causal effects. Future research using longitudinal panel and diary study designs can address issues of causal effects and causal direction, as well as allow the decomposition of associations involving work fatigue into between-person and within-person effects [85]. Second, the results may not generalize fully to the RCAF military; 16% of the sample was excluded from the present study because they completed the DWWS/DFS-FQ in French. Although a French version of the 3D-WFI is being developed, it was not ready for the present study. When complete, additional research should explore language invariance in the 3D-WFI. Third, work fatigue may have resulted in some nonresponse, leading to an underestimate of its prevalence. However, the prevalence estimates we report for this national military sample were substantial and higher than the prevalence rates for a national sample of civilian employees. In a related capacity, work fatigue could have influenced responses on the various measures for some individuals. However, we do not believe that exposure to work fatigue had a large impact on nonresponse or an overly corrupting impact on responses. Participants were able to complete the survey outside work hours and could choose to any day and time they were not fatigued. Nonetheless, the issue of whether and to what extent work fatigue may influence survey participation in or responses to organizational surveys is an interesting topic for future research. Fourth, all variables were obtained from self-reports. The use of self-reports is unavoidable for many of the constructs used in this study (e.g., role ambiguity, work fatigue, military morale, turnover intentions). Although it is typically assumed that common method variance (CMV) can inflate observed self-reported associations relative to the true population associations, CMV can deflated associations as well [86,87]. To minimize processes that are associated with CMV, such as consistency biases, demand characteristics, and social desirability biases, the study incorporated two procedural remedies to minimize the likelihood of CMV [88]. First, participants were informed that their responses would be confidential and no identifying information was collected from participants. Second, the measures used several different response scales and were separated across sections of a more extensive questionnaire.

## 8. Conclusions

Our findings show that physical, mental, and emotional work fatigue are highly prevalent among RCAF military personnel in a non-deployed setting. Moreover, all three types of work fatigue may be multi-determined and are associated with a broad set of adverse outcomes. Therefore, this study suggests that work fatigue is a critical issue among military personnel in non-deployed settings, and an important issue for military policy formation regarding health, work attitudes, safety, and performance. This study also showed that the 3D-WFI provides a psychometrically sound and valid assessment of work fatigue among military personnel. This measure can provide consistency in assessment and comparability of findings across future studies of work fatigue in the military.

## Figures and Tables

**Table 1:** Weighted Descriptive Statistics and Zero-Order Correlations for Study Variables.

Variables	*M*	*SD*	1	2	3	4	5	6	7	8	9	10	11	12	13	14	15	16
1. Physical fatigue	3.06	1.25	—															
2. Mental fatigue	3.08	1.28	0.79	—														
3. Emotional fatigue	2.50	1.35	0.71	0.80	—													
4. Role overload	3.85	1.38	0.48	0.55	0.49	—												
5. Role ambiguity	2.95	1.45	0.35	0.40	0.40	0.38	—											
6. Abusive supervision	1.35	0.73	0.23	0.26	0.32	0.24	0.25	—										
7. Distributive justice	3.66	1.24	−0.37	−0.40	−0.45	−0.38	−0.42	−0.35	—									
8. Interpersonal justice	3.86	0.94	−0.32	−0.32	−0.39	−0.23	−0.42	−0.52	0.47	—								
9. Organizational support	4.68	1.44	−0.39	−0.43	−0.47	−0.36	−0.47	−0.38	0.60	0.55	—							
10. Physical activity	9.25	4.78	−0.22	−0.17	−0.16	−0.05	−0.13	−0.01	0.09	0.08	0.07	—						
11. Sleep quantity	5.90	1.77	−0.31	−0.31	−0.31	−0.21	−0.15	−0.17	0.18	0.20	0.21	0.13	—					
12. Sleep quality	20.88	9.34	−0.46	−0.46	−0.48	−0.30	−0.24	−0.20	0.28	0.26	0.30	0.15	0.49	—				
13. Morale	3.32	0.99	−0.43	−0.49	−0.50	−0.31	−0.49	−0.25	0.42	0.43	0.57	0.24	0.19	0.34	—			
14. Workplace cognitive failure	1.99	0.57	0.43	0.47	0.46	0.32	0.38	0.23	−0.22	−0.29	−0.34	−0.11	−0.24	−0.34	−0.42	—		
15. Turnover intentions	2.48	1.19	0.35	0.41	0.41	0.34	0.36	0.23	−0.37	−0.29	−0.50	−0.12	−0.13	−0.29	−0.55	0.29	—	
16. Work–family conflict	4.03	1.76	0.49	0.52	0.48	0.64	0.36	0.27	−0.41	−0.27	−0.46	−0.04	−0.23	−0.36	−0.34	0.25	0.36	—

***Note.****N* = 1375. Weighted means, standard deviations, and correlations are reported. Correlations greater than |0.05| are significant at *p* < 0.05 (two-tailed).

**Table 2:** Measurement Invariance Testing.

Model	S-B χ^2^	*df*	CF	RMSEA	RMSEA 95% CI LLUL		CFI	SRMR	Model Comparison	ΔS-B χ^2^	Δ*df*	ΔCFI
**Sex**												
Single-group												
Male (*n* = 1175)	548.30	114	2.39	0.057	0.052	0.062	0.965	0.027				
Female (*n* = 200)	396.15	114	1.71	0.111	0.099	0.123	0.913	0.033				
Multiple-group invariance (*N* = 1375)												
1. Configural	969.71	228	2.05	0.069	0.064	0.073	0.956	0.028				
2. Weak	1009.53	243	1.99	0.068	0.063	0.072	0.955	0.028	1	19.53	15	−0.001
3. Strong	1045.88	258	1.95	0.067	0.062	0.071	0.954	0.028	2	24.24	15	0.000
4. Strict	1012.11	276	2.12	0.062	0.058	.066	.957	0.029	3	23.31	18	+.002
5. Strict + correlated residuals	1035.97	294	2.11	0.061	0.057	0.065	0.956	0.029	4	20.56	18	−0.001
**Age**												
Single-group												
18–34 years old (*n* = 416)	280.45	114	1.92	0.059	0.051	0.068	0.968	0.034				
35–54 years old (*n* = 803)	574.68	114	2.35	0.071	0.064	0.077	0.947	0.028				
55 years and older (*n* = 156)	200.58	114	1.70	0.070	0.054	0.085	0.969	0.021				
Multiple-group invariance (*N* = 1375)												
1. Configural	1120.70	342	1.99	0.070	0.066	0.075	0.956	0.029				
2. Weak	1182.96	372	1.91	0.069	0.065	0.073	0.954	0.031	1	29.32	30	−0.002
3. Strong	1239.34	402	1.87	0.067	0.063	0.072	0.952	0.031	2	42.29	30	−0.002
4. Strict	1229.28	438	2.09	0.063	0.059	0.067	0.955	0.033	3	55.34 *	36	+0.003
5. Strict + correlated residuals	1290.26	474	2.10	0.061	0.057	0.065	0.954	0.033	4	36.17 **	36	−0.001
**Military component**												
Single-group												
Regular Force (*n* = 1143)	547.52	114	2.33	0.058	0.053	0.063	0.965	0.026				
Primary Reserve (*n* = 232)	373.51	114	1.75	0.099	0.088	0.110	0.927	0.040				
Multiple-group invariance (*N* = 1375)												
1. Configural	946.12	228	2.04	0.068	0.063	0.072	0.957	0.029				
2. Weak	994.86	243	1.98	0.067	0.063	0.071	0.955	0.032	1	37.21**	15	−0.002
3. Strong	1027.82	258	1.95	0.066	0.062	0.070	0.954	0.031	2	24.68	15	−0.001
4. Strict	991.56	276	2.13	0.061	0.057	0.066	0.958	0.034	3	22.88	18	+0.004
5. Strict + correlated residuals	1041.94	294	2.14	0.061	0.057	0.065	0.956	0.034	4	51.34***	18	−0.002
**Military role**												
Single-group												
Aircrew (*n* = 282)	479.64	114	1.66	0.107	0.097	0.117	0.902	0.041				
Maintenance technician (*n* = 422)	255.63	114	2.00	0.054	0.045	0.063	0.970	0.038				
Other support role (*n* = 671)	440.12	114	2.15	0.065	0.059	0.072	0.962	0.019				
Multiple-group invariance (*N* = 1375)												
1. Configural	1163.33	342	1.93	0.072	0.068	0.077	0.953	0.031				
2. Weak	1234.05	372	1.86	0.071	0.067	0.076	0.951	0.033	1	47.18	30	−0.002
3. Strong	1290.09	402	1.82	0.069	0.065	0.074	0.949	0.034	2	39.75	30	−0.002
4. Strict	1270.35	438	2.08	0.064	0.060	0.069	0.953	0.034	3	59.07 **	36	+0.004
5. Strict + correlated residuals	1286.27	474	2.11	0.061	0.057	0.065	0.954	0.034	4	28.97	36	+0.001

***Note.****N* = 1375. Unweighted samples sizes and weighted confirmatory factor analyses are reported. *df* = degrees of freedom; CF = correction factor; S-B = Satorra–Bentler; RMSEA = root mean square error of approximation; CI = confidence interval; LL = lower limit, UL = upper limit; CFI = comparative fit index; SRMR = standardized root mean square residual. All model χ2 values are significant at *p* < 0.001. For Δχ^2^, * *p* < 0.05. ** *p* < 0.01. *** *p* < 0.001.

**Table 3:** Prevalence of Work Fatigue.

	Physical Fatigue			Mental Fatigue			Emotional Fatigue		
Variables	% ≥ Monthly	% ≥ Weekly	% Daily	% ≥ Monthly	% ≥ Weekly	% Daily	% ≥ Monthly	% ≥ Weekly	% Daily
**Overall sample** (*N* = 1375)	62.2	40.6	13.7	62.7	42.9	14.6	43.4	29.2	8.7
**Sex**									
Male (*n* = 1175)	62.4_a_	40.2_a_	12.6_b_	61.6_a_	41.2_b_	13.7_a_	42.1_b_	27.8_b_	7.9_a_
Female (*n* = 200)	60.8_a_	42.5_a_	20.6_a_	69.0_a_	53.8_a_	20.5_a_	51.3_a_	37.9_a_	13.5_a_
**Age**									
34 years and under (*n* = 416)	64.0_a_	38.7_a_	12.1_a_	65.3_a_	42.2_a_	14.5_ab_	43.9_ab_	28.4_ab_	7.9_a_
35-54 (*n* = 803)	63.5_a_	43.0_a_	15.8_a_	63.6_a_	45.7_a_	15.8_a_	45.3_a_	31.2_a_	9.7_a_
55 and over (*n* = 156)	48.3_b_	34.3_a_	9.0_b_	47.9_b_	29.3_b_	8.9_b_	30.4_b_	20.7_b_	5.8_a_
**Military Component**									
Regular Force (*n* = 1143)	65.3_a_	43.5_a_	14.8_a_	65.6_a_	46.0_a_	16.0_a_	46.0_a_	30.9_a_	9.5_a_
Primary Reserve (*n* = 232)	41.4_b_	21.6_b_	6.6_b_	42.6_b_	22.4_b_	5.6_b_	26.1_b_	18.0_b_	3.0_b_
**Military Role**									
Aircrew (*n* = 282)	65.4_a_	42.3_a_	10.9_b_	64.8_a_	41.7_a_	11.7_a_	38.4_a_	27.9_a_	7.8_a_
Maintenance technician (*n* = 422)	64.1_a_	40.5_a_	10.7_b_	63.3_a_	42.3_a_	13.1_a_	45.0_a_	28.8_a_	8.1_a_
Other support role (*n* = 671)	59.4_a_	40.0_a_	17.4_a_	61.3_a_	43.9_a_	17.0_a_	44.0_a_	30.0_a_	9.5_a_

***Note.*** Unweighted samples sizes and weighted percentages are reported. Percentages with differing subscripts within columns are significantly different at *p* < 0.05.

**Table 4:** Regression of Work Fatigue on Predictor Variables.

Predictors	Physical Fatigue		Mental Fatigue		Emotional Fatigue	
	*r*	β	*r*	β	*r*	β
**Work demands**						
Role overload	0.48	0.29***	0.55	0.35***	0.49	0.26***
Role ambiguity	0.35	0.05	0.40	0.10***	0.40	0.07*
Abusive supervision	0.23	−0.01	0.26	0.01	0.32	0.05
**Work resources**						
Distributive justice	−0.37	−0.07*	−0.40	−0.06*	−0.45	−0.11**
Interpersonal justice	−0.32	−0.07*	−0.32	−0.02	−0.39	−0.08*
Perceived organizational support	−0.39	−0.09*	−0.43	−0.12**	−0.47	−0.12***
**Personal resources**						
Physical activity	−0.22	−0.14***	−0.17	−0.09***	−0.16	−0.07**
Sleep quantity	−0.31	−0.06	−0.31	−0.05	−0.31	−0.04
Sleep quality	−0.46	−0.25***	−0.46	−0.24***	−0.48	−0.26***
***R*^2^**		0.40***		0.46***		0.46***

***Note.****N* = 1375. Weighted regression results are reported. *r* = zero-order correlation; β = standardized regression coefficient. All correlations are significant at *p* < 0.001. *p < 0.05. **p < 0.01. ***p < 0.001.

**Table 5:** Regression of Outcomes on Work Fatigue.

	Morale		Workplace Cognitive Failure		Turnover Intentions		Work–Family Conflict	
Predictors	*r*	β	*r*	β	*r*	β	*r*	β
Physical fatigue	−0.43	−0.05	0.43	0.10*	0.35	0.02	0.49	0.19***
Mental fatigue	−0.49	0.22***	0.47	0.21***	0.41	0.22***	0.52	0.25***
Emotional fatigue	−0.50	0.29***	0.46	0.22***	0.41	0.23***	0.48	0.15**
***R*^2^**		0.27***		0.24***		0.19***		0.29***

***Note.****N* = 1375. Weighted regression results are reported. *r* = zero-order correlation; β = standardized regression coefficient. All correlations are significant at *p* < 0.001. *p < 0.05. **p < 0.01. ***p < 0.001.
